# Higher Muscle Damage Triggered by Shorter Inter-Set Rest Periods in Volume-Equated Resistance Exercise

**DOI:** 10.3389/fphys.2022.827847

**Published:** 2022-02-28

**Authors:** Gilmar Weber Senna, Estélio Henrique Martin Dantas, Estevão Scudese, Paula Paraguassú Brandão, Vitor A. Lira, Matheus Baffi, Luiz Claudio Pereira Ribeiro, Roberto Simão, Ewan Thomas, Antonino Bianco

**Affiliations:** ^1^Sports Science and Exercise Laboratory (LaCEE), Petrópolis Catholic University, Petrópolis, Brazil; ^2^Nursing and Biosciences Post-graduation Program, Doctorate of Federal University of State of Rio de Janeiro, Rio de Janeiro, Brazil; ^3^Biosciences Laboratory of Human Movement (LABIMH), Tiradentes University, Aracaju, Brazil; ^4^Department of Health and Human Physiology, The University of Iowa, Iowa City, IA, United States; ^5^School of Physical Education and Sports, Federal University of Rio de Janeiro, Rio de Janeiro, Brazil; ^6^Sport and Exercise Sciences Research Unit, Department of Psychology, Educational Science and Human Movement, University of Palermo, Palermo, Italy

**Keywords:** cytokines, creatine kinase, L-Lactate Dehydrogenase, leukocytes, resistance training, physical fitness

## Abstract

**Objectives:**

The aim of the manuscript was to analyze the effects of two rest periods between volume-equated resistance exercise (RE) on inflammatory responses (cytokines and leukocyte) and muscle damage.

**Methods:**

Ten trained men (26.40 ± 4.73 years, 80.71 ± 8.95 kg, and 176.03 ± 6.11 cm) voluntarily participated in training sessions consisting of five sets of 10 reps performed at 10-RM on (1) the barbell bench press followed by (2) leg press, with either 1- or 3-min rest between sets and exercises. Circulating concentrations of different biomarkers was measured before (Pre), and after 3 h (excepted for cytokines), 6, 12, and 24 h from exercise. The rate of perceived exertion (RPE) was recorded after each set on both planned visits.

**Results:**

We found greater increases triggered by the 1-min rest period in Creatine Kinase (CK), occurring from 12 to 24 h post-exercise compared to the 3-min rest condition. A significant increase in the 1-min rest condition was also observed in the total number of leukocytes, neutrophils, and monocytes. The 1-min rest period also triggered increases compared to baseline in pro-inflammatory cytokines [Interleukin 1 beta (IL-1β), *p* = 0.004; tumor necrosis factor α (TNF-α), *p* = 0.01; and granulocyte-macrophage colony-stimulating factor (GM-CSF), *p* = 0.01], which were more evident after 6 and 12 h post-exercise. Similarly, increases in anti-inflammatory cytokines [Interleukin 5 (IL-5), *p* = 0.01; Interleukin 6 (IL-6), *p* = 0.01; and Interleukin 10 (IL-10), *p* = 0.01] at all time-points were observed.

**Conclusion:**

Our results indicate that a 1-min rest condition in volume-equated RE promoted greater overall muscle tissue damage with a longer duration of the inflammatory processes compared to a 3-min rest.

## Introduction

Regarding general neuromuscular health, resistance exercise (RE) is considered the most efficient method for enhancing muscular strength, power, and muscular endurance (American College of Sports Medicine, 2009), triggering multiple benefits with numerous positive outcomes evident in the literature (Garber et al., 2011). However, the beneficial effects of resistance exercise can be influenced by exercise selection, exercise order, intensity, number of sets, and resting interval between sets (American College of Sports Medicine, 2009). Modulation of such parameters determines specific adaptations toward increases in strength or hypertrophy (Borde et al., 2015; Schoenfeld et al., 2019).

In particular, regarding modulation of inter-set lengths, evidence suggests that either shorter or longer inter-set duration (within a range of 60 s to 3 min) may promote muscle hypertrophy (Grgic et al., 2017), while longer rests (usually 3 min or longer) seem to be more beneficial for strength development (de Salles et al., 2009). Therefore, it is still not clear which mechanisms mediated by inter-set length may determine hypertrophic adaptations. Previous studies indicated that differences in resting intervals between sets may independently influence the repetition performance (Senna et al., 2011, 2016; Scudese et al., 2015; Matos et al., 2021), as well as promote changes in neuromuscular (Willardson and Burkett, 2008), endocrine (Scudese et al., 2016), cardiorespiratory (Ratamess et al., 2007), and even immune responses to the exercise session (Gerosa-Neto et al., 2016; Rossi et al., 2016).

During RE sessions, the muscular tissue is exposed to micro-injury (which occurs after concentric and eccentric contractions, with greater damage observed after eccentric exercise) (Markus et al., 2021). Exercise-induced muscle damage (EIMD) has been mainly observed in the sarcolemma, cytoskeleton, and contractile elements of the muscle. Despite EIMD may have short-term (24–48 h) detrimental effects on muscle function, due to pain and inflammation, it is thought that the associated muscle inflammation promotes increased protein turnover, which may lead to long-term adaptations (Schoenfeld, 2012; Fukada et al., 2020). In particular, short-inter-set lengths (60 s or below) are seen to promote greater muscle damage than longer rest periods (3 min or above) (Machado and Willardson, 2010) and acutely increase anabolic hormonal levels (Henselmans and Schoenfeld, 2014). EIMD also increases the expression of both pro and anti-inflammatory cytokines of local muscle tissue (Calle and Fernandez, 2010; Minari and Thomatieli-Santos, 2021). Modulation of genetic muscle regulators and activation of muscle satellite cells, mediated by different anti-inflammatory cytokines, may be one of the major factors that lead to the regeneration or even hypertrophy of muscle tissue (Peake et al., 2006). However, muscle damage and inflammatory response derived from RE are still not completely elucidated and might play a key role regarding the physiological mechanism related to RE adaptations (Spiering et al., 2008; Margaritelis et al., 2021).

Research has indicated that once a tissue is damaged, similar increases in biochemical markers occur as a consequence of the extravasation of the content of the damaged cells, despite different stimuli are provided (Rodrigues et al., 2010; Evangelista et al., 2011; Machado et al., 2012). Increases which also occur after acute bouts of high-intensity interval training [increases observed in Interleukin 6 (IL-6), Interleukin 10 (IL-10), and tumor necrosis factor α (TNF-α); Gerosa-Neto et al., 2020]. After muscle damage occurs, an increase in leukocyte recruitment to the bloodstream is observed as an attempt to reverse the general breakdown (Kilgore et al., 2002; Fatouros and Jamurtas, 2016) due to the process of repair and tissue remodeling (Raastad et al., 1985).

Changes in the hemodynamic profile driven by mechanical stimuli, promote increased sympathetic action with release of catecholamines during exercise. These appear to be an important factor driving the elevation of leukocyte concentration, promoting the cellular recruitment of lymphoid organs and endothelium to the blood by mechanical loading (Pedersen and Hoffman-Goetz, 2000).

Consequently, different cytokines have effects on the mobilization of neutrophils from the bone marrow to the blood, increasing the leukocyte count beyond the patterns presented immediately at the end of an exercise session (Pedersen and Hoffman-Goetz, 2000), thereby triggering a second phase increase in cell types. This physiological cascade of events that characterizes the immune system profile occurs at different time-point peaks that may not immediately fall after the exercise (Mayhew et al., 2005; Chatzinikolaou et al., 2010). Although the general inflammatory response is vastly clinically studied, little is known between the immune system response related to modulation of RE parameters, as different rest intervals between sets (Mayhew et al., 2005). Few experiments have attempted to elucidate the behavior of this process by analyzing the biomarkers of tissue damage, leukocyte profile, and cytokine expression of pro and anti-inflammatory action after RE performed with different intervals between sets (Rodrigues et al., 2010; Evangelista et al., 2011; Machado et al., 2012; Rossi et al., 2016). Therefore, the purpose of this study was to analyze the effects of two distinct rest period lengths (1 and 3 min, which are typically used in hypertrophy or strength protocols, respectively) between RE sets of equalized volume on muscle tissue damage [CK and Lactate Dehydrogenase (LDH)] and inflammatory biomarkers responses.

## Materials and Methods

### Experimental Approach to the Problem

Inter-set lengths represent a crucial aspect of modern resistance training. Different interest lengths are used in hypertrophy or strength training. In our study, inter-set length in volume-equated RE protocols represented the primary independent variable. Biomarkers of muscle damage and inflammation were dependent variables on the experimental time exposure. A cross-over research design was carried out due to the lack of a comparison/control group; therefore, intra-group comparisons were used between the two conditions.

### Subjects

Ten trained men with at least 1 year of consistent RE experience were selected to participate (26.40 ± 4.73 years, 80.71 ± 8.95 kg, 176.03 ± 6.11 cm, 9.86 ± 3.25% body fat, Bench Press (BP) relative strength: 1.27 ± 0.27 kg.kg^-1^ of body mass, Leg press (LP) relative strength: 3.66 kg.kg^−1^ of body mass.). With the intent to standardize the subject selection, the following inclusion criteria were adopted: (a) engaged in at least 1 year of resistance training with a frequency of at least four times per week, with session duration approximately 1 h; (b) non-usage of any ergogenic substance that would enhance repetition performance; (c) no acute or chronic injuries that would affect BP and LP performance; (d) no usage of medicines that may alter metabolism; (e) no pre-established endocrine diseases, cardiopathies, arterial hypertension, uncontrolled asthma, and any musculoskeletal conditions that could serve as an intervening factor in the practice of the activity (osteoarthritis, recent fracture, tendinitis, and use of prosthesis) and immune system alterations; and (f) agreement to not engage in any type of intense or structured physical activity throughout test days. Before data collection, all subjects answered “no” to all PAR-Q questions (Shephard, 1988). The study procedures were approved by the Federal University of State of Rio de Janeiro ethics committee (CAAE 63803717.2.0000.5285). Besides, all subjects read and signed a consent form after being informed of the testing procedures according to the Declaration of Helsinki.

### Ten Maximum Repetitions Test

After two familiarization sessions, subjects completed the 10 maximum repetitions (10-RM) test sessions for bench press (BP) and leg press (LP), with each session separated by 72 h. Each familiarization session consisted in simulating the testing protocols for both exercises (BP and LP) without the use of additional weight. For instance, participants were instructed on how they should perform exercise techniques with proper execution, in order to minimize the chance of injury, and they were also asked about their subjective rate of perceived effort intending to train their ratings when the actual testing sessions began. The 10-RM testing procedures followed the recommendations of Baechle and Earle (2000). To minimize testing errors, the following strategies were adopted for all exercise sessions: (a) standardized instructions were provided to each subject; (b) proper BP and LP technique was explained to all subjects, and compliance with this technique was verified for each repetition by an experienced investigator; (c) body positioning on the BP and LP was held constant and maintained throughout the whole experiment; and (d) verbal encouragement was applied during all testing sessions to encourage maximum effort from each subject; and € the weight of the barbell and all plates were measured with a precision scale.

For the 10-RM determination, a progressive loading strategy was adopted, so that the minimum increase of weight was a total of 2 kg for BP, and 5 kg of LP after every successful attempt. After the warm-up, a maximum of five attempts was permitted for each 10-RM testing session, with a minimum of 10-min recovery between the lifting attempts.

Excellent test–retest reliability was verified by the intraclass correlation coefficient (BP, *r* = 0.99; LP, *r* = 0.99) for the 10-RM test. In addition, a paired student *t* test did not show any significant differences between test–retest 1RM loads (BP, *p* = 0.16; LP, *p* = 0.10).

### Muscle Tissue Damage Biomarkers and Leukocyte Count

All muscle tissue damage biomarkers and leukocyte count were verified at pre-exercise, 3, 6, 12, and 24 h post-exercise, with the blood samples being collected by venipuncture from an antecubital vein for following the determination of the concentrations of circulating CK and LDH, Leucocyte counts and its sub-types. Biochemical analysis was performed using commercial kits [lots of the CK: 182906-01 (Roche); LDH: 00202541 (Roche); Reagent Cellpack: p7066; Stromatolyser 4DL: p6014; and Sulfolyser: p6009] specific to humans in an automatic device [Cobas E601 (Roche)]. For CK and LDH, we used the electrochemiluminescence method. The hematological analysis was performed immediately after the collection through automated analysis (KX-21N, Sysmex) using the tubes containing the blood collected over the several distinct time-points by the photometric method.

### Cytokines Concentrations

The cytokines levels for interleukin (IL)-1β, IL-5, IL-6, IL-10, tumoral necrosis factor alfa (TNF-α), and granulocyte-macrophage colony-stimulating factor (GM-CSF) in the serum were determined by in an automatic device multiplex (Luminex™ 100/200 model) flow cytometry-based. The cytokine analysis was performed using commercial kits to magnetic cytokine human panel [Thermos-Fisher Scientific™ (LHC0001M)]. Assays performed by this system provide comparable intra- and inter-assay precision with typical coefficient of variation values of <10% over the analyzed concentration range. Blood samples were collected at Pre, 6, 12, and 24 h post-exercise for this analysis. All samples for each participant were collected considering the timing of the workout session. After data collection, the IL-10/TNF-α ratio was calculated. For all assessments, the analytical sensitivity was <0.5 pg./ml; except for GM-CSF that the analytical sensitivity was <0.05 pg./ml. A 1:3 dilution between the curve points was adopted for this experiment.

### Rate of Perceived Exertion Procedure

The OMNI RE scale (Lagally and Robertson, 2006) was implemented to obtain the (RPE) values. Subjects were previously familiarized and asked to choose a number based on their perceived exertion or subjective intensity of effort, strain, discomfort, and fatigue experienced during the exercise session. Immediately after each exercise set, subjects were asked to identify their RPE to provide a subjective measure of the exertion level.

### Resistance Exercise Session

**Figure 1:**
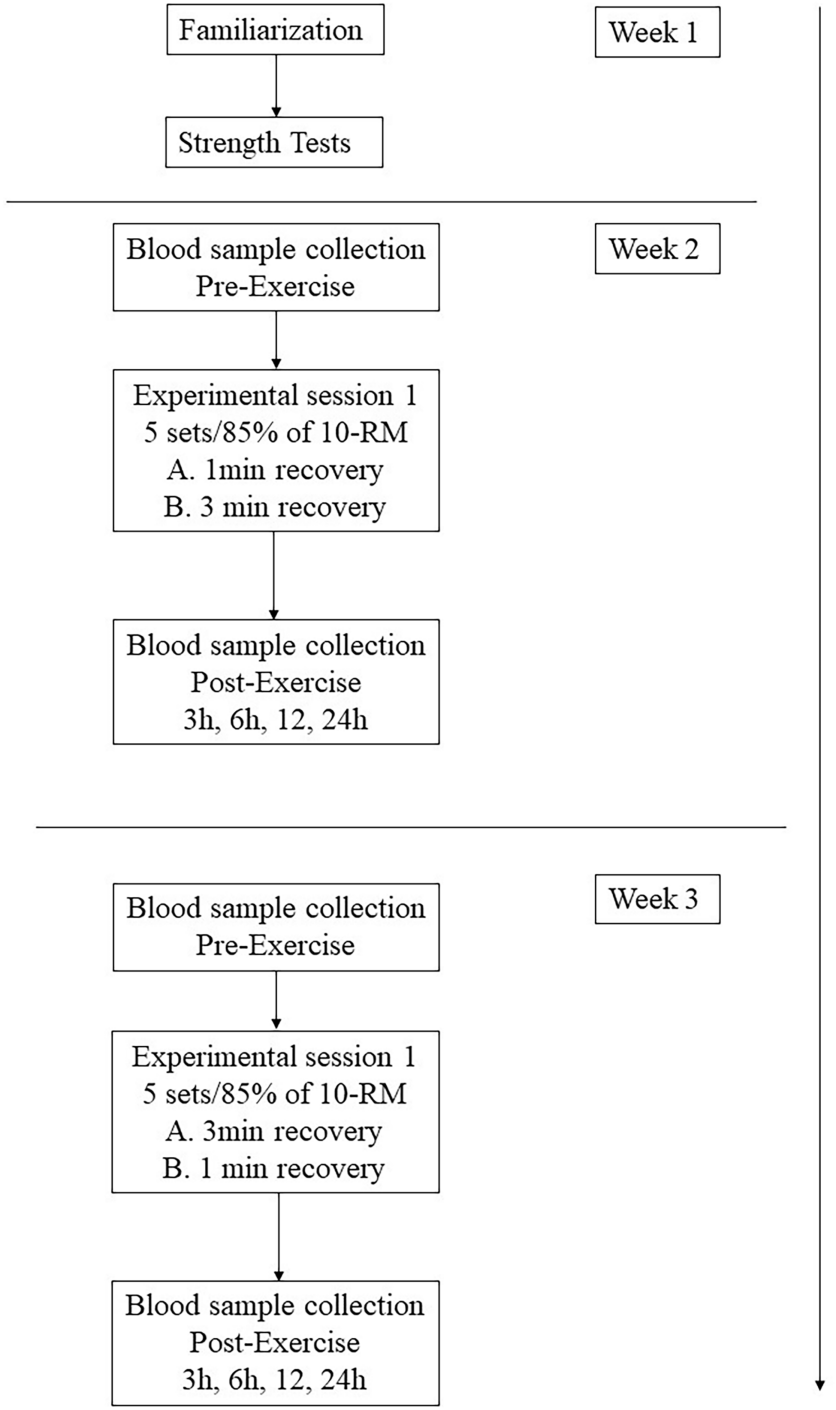
Flowchart of the experimental approach to the problem.

One week after the last 10-RM test session, subjects completed the first workout session, which consisted of the execution of the BP and subsequently the LP exercise. The starting order of inter-set length conditions (1- or 3-min rest between sets) was counterbalanced and assigned through randomization. One week later, subjects completed the same workout protocol, with the remaining resting condition; in this manner, each participant performed both exercise protocols (Figure 1).

Before each workout, a standardized warm-up was performed consisting of two sets of 12 repetitions with 40% of a 10-RM load that was pre-estimated based on each subject’s workout routine. A 2-min rest interval was observed after the warm-up and before the workout sets. No attempt was made to control the repetition velocity; however, subjects were required to use a smooth and controlled movement throughout range of motion (Senna et al., 2011). In order to avoid any potential confounding effects of the circadian cycle responses, all workout sessions were conducted between 06:00 a.m. and 08:00 a.m. Also, the time of data collection for a particular subject was held constant for each workout session (Scudese et al., 2016).

### Nutritional Control

On the day before the first workout session, subjects recorded their dietary intake with an alimentary intake frequency questionnaire (AIFQ). After this verification, we observed that the participants ingested 2771.50 ± 135.10 kcal on the day before the first workout session, specifically: (a) carbohydrates: 1274.89 ± 71.60 kcal; (b) protein: 914.60 ± 44.58 kcal; and (c) fat: 582.02 ± 28.37 kcal. Subjects received a copy of their diet record and were instructed to reproduce, as strictly as possible, this recorded dietary pattern on the day before the second workout. Subjects arrived at the laboratory after an overnight fast and were fed with a standard breakfast 1 h before each workout session in order to standardize the acute nutritional status. More specifically, the breakfast macronutrient distribution consisted of 40 g of carbohydrates, 8 g of protein, 6 g of fat, and 5 g of fiber, and totaling 256 kcal of energy content.

The standardized meal was prepared to provide recommended amounts of macronutrient proportions according to the ACSM position stand on nutrition and athletic performance (Thomas et al., 2016). After the training sessions, subjects were oriented to maintain their regular nutritional habits *via* professional nutritionist guidance and were asked to avoid any extrapolation that might exacerbate the inflammatory response. Besides, in order to ensure that subjects arrived in a euhydrated state, they were instructed to ingest 5–7 ml of water per kilogram of bodyweight immediately on awakening on workout days (Thomas et al., 2016).

### Statistical Analysis

Variables are presented as means ± SD. RPE is presented according to the median and interquartile range. A two-way ANOVA with repeated measures on both factors (rest condition vs. time points) was conducted for each blood analysis and ratio of the included variables. Fisher’s least significant difference was used to identify pairwise differences when applicable. Effect sizes (ESs) for changes from Pre to 3, 6, 12, and 24 h were calculated. In order to interpret the magnitude of the ESs, the limits proposed by Cohen were adopted (Cohen, 1988). For blood concentrations data, the area under the curve (AUC) was calculated using the trapezoidal method and compared between rest conditions using a paired mean *t* test. The Friedman test was used to investigate non-parametric data for RPE to compare differences in values between the distinct sets and rest protocols. When appropriate, Dunn’s *post hoc* analysis was applied for multiple comparisons. Additionally, the Wilcoxon test was used for comparisons between RPE values resulting from different rest intervals. The level of significance assumed was *p* ≤ 0.05. All statistics were performed *via* SPSS software, version 22.0 (IBM, Inc., United States).

## Results

### Muscle Tissue Damage Biomarkers

**Figure 2:**
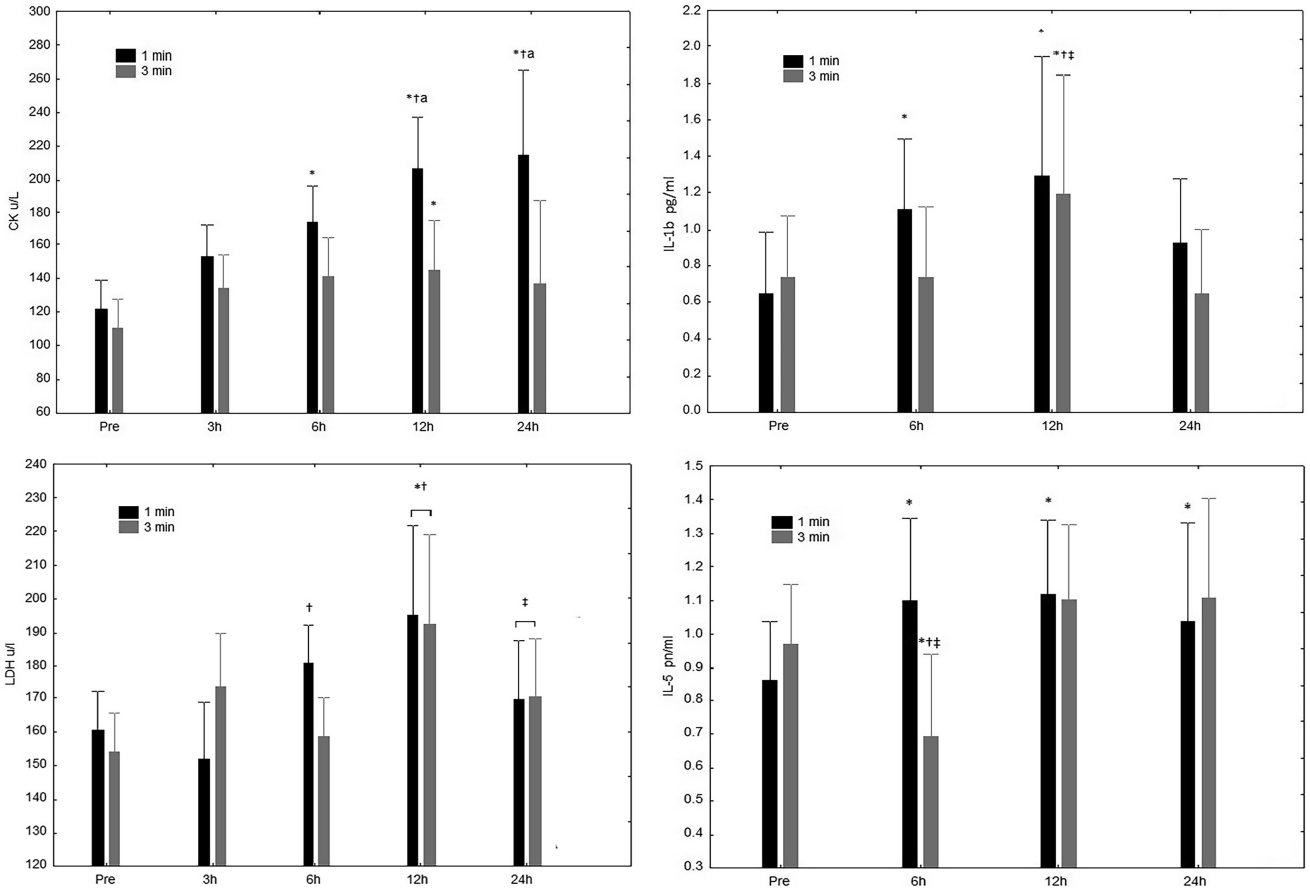
CK/ul Creatine Kinase (CK) concentrations at pre-exercise (Pre), 3, 6, 12, and 24 h after completing five sets of bench press and leg press exercises with 1 or 3 min of rest between sets. LDH/ul L-Lactate Dehydrogenase (LDH) concentrations at pre-exercise (Pre), 3, 6, 12, and 24 h after completing five sets of bench press and leg press exercises with 1 or 3 min of rest between sets. ^*^Significant difference to Pre (*p* ≤ 0.05); ^†^Significant difference to 3 h (*p* ≤ 0.05); ^‡^Significant difference to 12 h (*p* ≤ 0.05). ^a^Significant difference compared to 3-min rest condition (*p* ≤ 0.05); IL-1b pg/ml Interleukin-1 β (IL-1β) concentrations at pre-exercise (Pre), 3, 6, 12, and 24 h after completing five sets bench press and leg press exercises with 1 or 3 min of rest between sets. IL-5 pn/ml Interleukin-5 (IL-5) concentrations at pre-exercise (Pre), 3, 6, 12, and 24 h after completing five sets of bench press and leg press exercises with 1 or 3 min of rest between sets. ^*^Significant difference to Pre (*p* ≤ 0.05); ^†^Significant difference to 6 h (*p* ≤ 0.05); ^‡^Significant difference to 24 h (*p* ≤ 0.05); and ^a^significant difference compared to 3-min rest condition (*p* ≤ 0.05).

A significant interaction (rest condition vs. time-point) for CK concentration (Figure 2) was observed (*p* = 0.02). Specifically, the main effect for time (*p* < 0.01), revealed increases after 6, 12, and 24 h in the 1-min rest condition; increases were observed only after 12 h in the 3-min rest condition. Regarding the main effect between conditions (*p* = 0.01) greater increases were observed after only after 12 h. The AUC differed (*p* < 0.01) between the 1-min (4572.4 ± 1169.5 u/L.h^−1^) and 3-min (3330.1 ± 715.9 u/L.h^−1^) condition (Figure 2). ESs appointed larges increases in CK magnitudes from 3 to 24 h time-points for 1-min (3 h, 1.15; 6 h, 1.96; 12 h, 3.19; and 24 h, 3.48) compared to the 3-min condition (3 h, 0.97; 6 h, 1.04 12 h, 1.07; and 24 h, 1.00).

No significant interaction for LDH concentration (Figure 2) was observed (rest condition vs. time-point). More specifically, both rest protocols resulted in significant increases in LDH after 12 h; while a reduction was observed after 24 h regardless of the condition. For the main effect between conditions, no significant difference (*p* = 0.79) was observed.

### Leucocyte Count

For the leucocyte count, we found a significant interaction (rest condition vs. time-point; *p* < 0.01). For instance, the main effect regarding time (*p* < 0.01), revealed that for the 1-min rest condition increases were present at 3, 6, and 12 h; and significant reductions occurred between 12 and 24 h post exercise. For the main effect between rest conditions, no difference (*p* = 0.91) was found. The ESs presented larger increases in the total number of leukocytes for the 1-min rest condition. All values are reported in Table 1.

**Table 1:** Total number of leukocytes, neutrophils, lymphocytes, monocytes area under the curve (AUC), and effect size (ESs) at pre-exercise (Pre), 3 h (3 h), 6 h (6 h), 12 h (12 h), and 24 h (24 h) after completing five sets of bench press and leg press exercises with 1- or 3-min of rest between sets.

Rest condition	Pre	3 h	6 h	12 h	24 h	AUC	ESs (d) value for AUC
Total number of leucocytes (mm^3^)						
1 min	5901.9 ± 966.9	6664.1 ± 1034.1^*^	6816.9 ± 1292.4^*^	7879.5 ± 915.3^*^^#^^†^	6432.4 ± 1290.1^‡^	169031.0 ± 23175.4	0.32
ESs (d)	-	0.79	0.95	2.05	0.55		
3 min	6555.9 ± 1286.3	6421.61 ± 1256.2	6676.9 ± 1,394,7	6951.8 ± 1885.0	6643.6 ± 1087.9	161574.0 ± 33753.5	
ESs (d)	-	−0.10	0.09	0.30	0.06		
Neutrophils count (mm^3^)							
1 min	3230.7 ± 794.6	3980.3 ± 876.8^*^	3912.6 ± 909.7^*^	4361.0 ± 1057.3^*^ ^†^	3232.8 ± 1008.5^#^^†^^‡^	93039.5 ± 20466.7	0.12
ESs (d)	-	0.94	0.86	1.42	0.00		
3 min	3645.7 ± 1008.7	3799.5 ± 1101.4	3807.1 ± 1215.7	3955.2 ± 1124.8	3504.6 ± 840.4	90624.2 ± 24203.5	
ESs (d)	-	0.85	0.86	0.91	0.74		
Lymphocytes count (mm^3^)							
1 min	1885.6 ± 470.5	1827.2 ± 473.9	2126.1 ± 193.5	2583.1 ± 444.9^*^ ^#^ ^†^	2367.0 ± 604.8^*^ ^#^	55328.4 ± 8997.3	0.390
ESs (d)	-	−0.12	0.51	1.48	1.02		
3 min	255.9 ± 458.7	1883.3 ± 360.6	2141.8 ± 382.2^#^	2158.9 ± 765.5	2335.5 ± 413.2^#^	51816.1 ± 10326.1	
ESs (d)	-	−0.38	0.19	0.22	0.61		
Monocytes count (mm^3^)							
1 min	517.1 ± 133.8	528.5 ± 80.2	543.2 ± 75.3	639.1 ± 94.2^*^ ^#^ ^†^	542.5 ± 133.2^‡^	13812.3 ± 2113.3	0.005
ESs (d)	-	0.09	0.21	0.91	0.19		
3 min	597.7 ± 75.2	566.8 ± 89.1	574.2 ± 89.9	571.3 ± 63.7	579.7 ± 96.6	13801.7 ± 1594.8	
ESs (d)	-	−0.41	−0.31	−0.35	−0.24		

* Significant difference to Pre (*p* ≤ 0.05);

# Significant difference to 3 h (*p* ≤ 0.05);

† Significant difference to 6 h (*p* ≤ 0.05);

‡ Significant difference to 12 h (*p* ≤ 0.05).

For neutrophils count, a significant interaction (rest condition vs. time-point; *p* = 0.03) was observed. Significant increases were present at 3, 6, and 12 h; while a reduction was present after 24 h. No significant differences (*p* = 0.98) were found between conditions. All values are reported in Table 1.

No significant interaction (rest condition vs. time-point) was evinced in lymphocytes count (*p* = 0.09). All values are reported in Table 1.

A significant interaction (rest condition vs. time-point; *p* < 0.01) for the monocytes count was found. More specifically, the for 1-min rest condition there was a significant increase after 12 h and a reduction after 24 h (*p* = 0.03). The main effect between conditions however did not show any significant difference (*p* = 0.50). All values are reported in Table 1.

### Cytokines Concentrations

A significant interaction was observed for IL-1β concentration for time (Figure 2; but not rest condition vs. time-point; *p* = 0.43). The 1-min rest protocol resulted in significant increases in Il-1β after 6 and 12 h; the 3-min rest interval showed an increase only after 12 h. No significant interaction was found between conditions.

For IL-5 concentration (Figure 2), a significant interaction effect was observed (rest condition vs. time point; *p* < 0.01). Significant increases in IL-5 were observed after 6, 12, and 24 h in the 1-min condition; decreased values were observed after 6 h for the 3-min rest interval. No significant interaction (*p* = 0.65) was seen between conditions.

**Figure 3:**
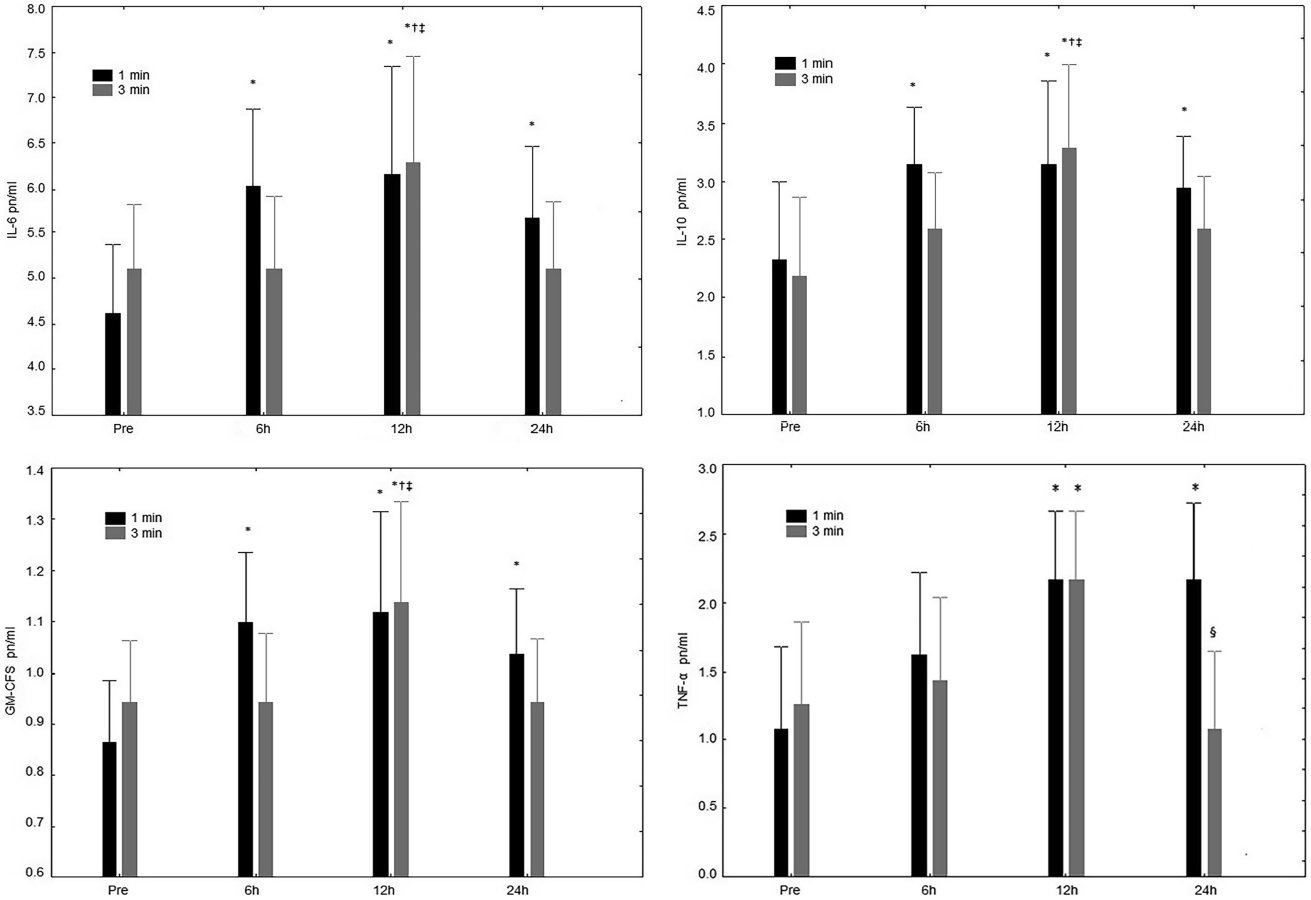
IL-5 pn/ml Interleukin-6 (IL-6) concentrations at pre-exercise (Pre), 3, 6, 12, and 24 h after completing five sets of bench press and leg press exercises with 1 or 3 min of rest between sets. IL-10 pn/ml Interleukin-10 (IL-10) concentrations at pre-exercise (Pre), 3, 6, 12, and 24 h after completing five sets of bench press and leg press exercises with 1 or 3 min of rest between sets. GM-CFS pn/ml Granulocyte-macrophage colonystimulating factor (GM-CSF) concentrations at pre-exercise (Pre), 3, 6, 12, and 24 h after completing five sets of bench press and leg press exercises with 1- or 3-min of rest between sets. TNF-α pn/ml Tumoral necrosis factor (TNF-α) concentrations at pre-exercise (Pre), 3, 6, 12, and 24 h after completing 5 sets of bench press and leg press exercises with 1- or 3-min of rest between sets. ^*^Significant difference to Pre (*p* ≤ 0.05); ^†^Significant difference to 6 h (*p* ≤ 0.05); ^§^Significant difference to 12 h (*p* ≤ 0.05); and ^‡^Significant difference to 24 h (*p* ≤ 0.05).

For IL-6 concentration (Figure 3), a significant interaction was observed (rest condition vs. time point; *p* = 0.04). Significant increases in IL-6 were present after 6, 12, and 24 h in the 1-min condition, while only after 12 h increases were observed in the 3-min rest interval. No significant interaction (*p* = 0.65) was seen between conditions.

Differences in IL-10 concentration (Figure 3), were not observed (rest condition vs. time point; *p* = 0.27). However, significant increases in Il-10 after 6, 12, and 24 h were present following the 1-min condition; and for the 3-min rest interval only after 12 h compared to all other time-points. No significant interaction (*p* = 0.65) was observed between conditions.

For GM-CSF concentration (Figure 3), a significant interaction (rest condition vs. time point) was observed (*p* = 0.04). More specifically, a main effect for time (*p* < 0.01), was observed after 6, 12, and 24 h in the 1-min condition, while an increase was present only after 12 h following the 3-min rest interval.

For TNF-α concentration (Figure 3), there were no significant interactions (rest condition vs. time point; *p* = 0.13). However, regarding time in the 1-min rest increases were observed after 12 and 24 h; while differences were present after the 3-min condition only after 12 h. No significant difference was present between rest conditions (*p* = 0.10).

**Figure 4:**
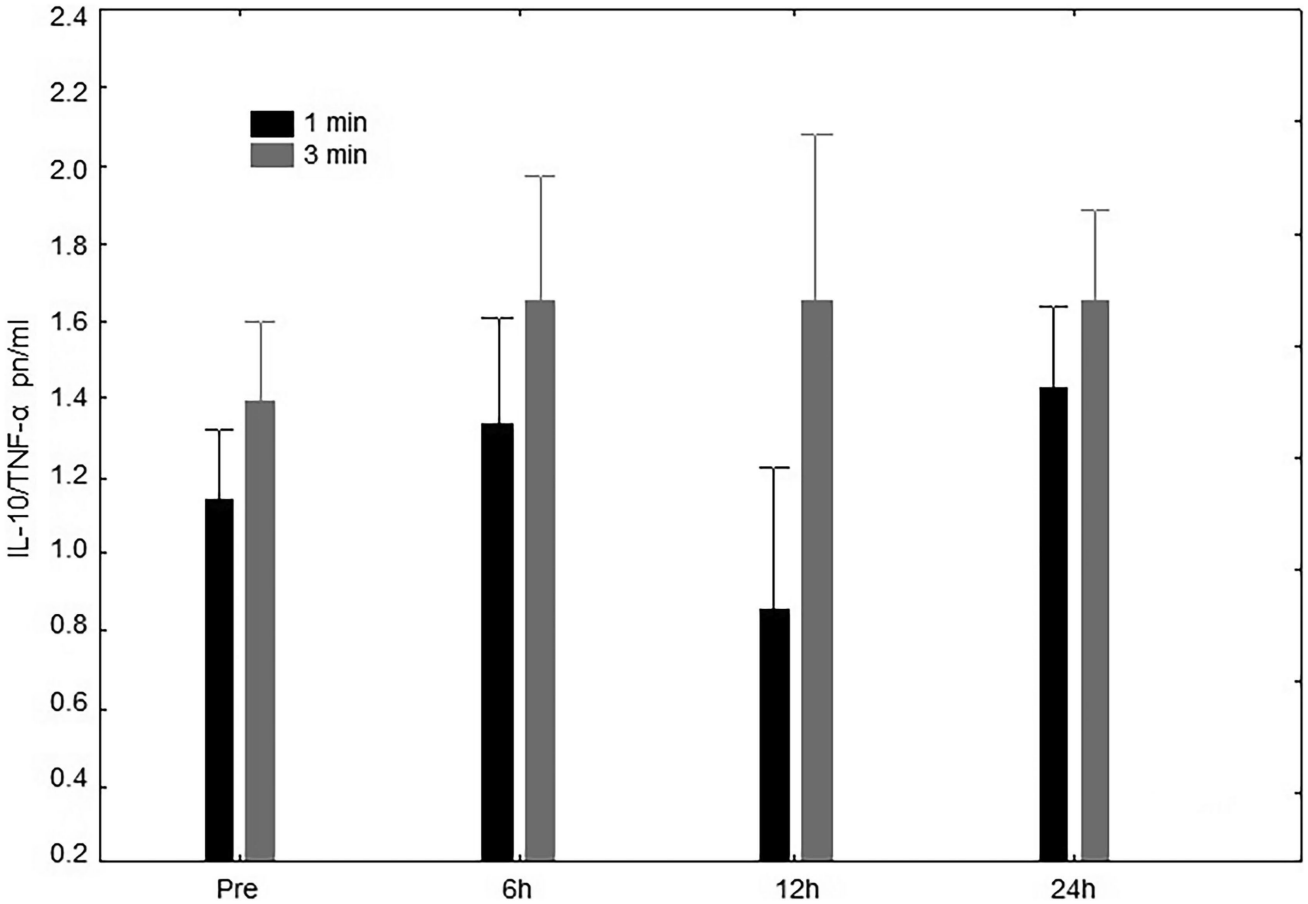
IL-10/TNF-α pn/ml Interleukin-10 and tumoral necrosis factor-α ratio (IL-10/TNF-α) concentrations at pre-exercise (Pre), 3, 6, 12, and 24 h after completing five sets of bench press and leg press exercises with 1- or 3-min of rest between sets.

For IL-10/TNF-α concentration, there was no significant interaction (rest condition vs. time point; *p* = 0.84). More specifically, no difference neither for time (*p* = 0.21) nor condition (*p* = 0.40) were observed (Figure 4). However, the AUC did differ (*p* = 0.03) between the 1-min (41.50 ± 9.18 pn/ml.h^−1^) and 3-min (26.98 ± 10.70 pn/ml.h^−1^) rest conditions.

### Rate of Perceived Exertion

Significantly higher values of post-set RPE were evident from the third set for the shorter 1-min rest condition compared to the first set, and only from the fourth set for the more extended 3-min rest condition compared to the first set. Additionally, significant differences were also observed in post-set RPE values, from the second set between conditions (SH, *p* < 0.01; LP, *p* < 0.01). All RPE data are presented in Table 2.

**Table 2:** Rating of perceived exertion (RPE). Post-set values for both exercises for the 1- and 3-min rest conditions [median (25%–75%)].

	Set 1	Set 2	Set 3	Set 4	Set 5
**Bench press**
1-min	4 (3–4)	5 (5–5.75)	7 (6.25–7.5)^*^	8 (8–8.5)^*^ ^#^	9 (9–9.5)^*^ ^#^
3-min	4 (3.25–4)	4.25 (4–4.5)^a^	4.75 (4.5–5)^a^	6 (5–6.5)^*^^a^	7 (5–7)^*#^^a^
**Leg press**
1-min	7 (7–7.5)	8 (8–8)	9 (8–9)^*^	9 (9–9)^*^	10 (9.5–10)^*^ ^#^
3-min	6.25 (5–7)	7 (5.5–7.5)^a^	7 (6–8)^a^	7.5 (6.5–8.5)^*#^^a^	7.5 (7–8.5)^*#^^a^

* Significant difference compared to Set 1;

# Significant difference compared to Set 2;

a Significant difference compared to 1-min rest condition.

## Discussion

Our key findings regard the muscle tissue damage (CK) triggered by the shorter inter-set length (1-min) observed from the early 12 h to the 24 h post-exercise window, compared to the 3-min rest condition. Increased muscle damage contributed to the increases observed in the total number of leukocytes, neutrophils (3–12 h post-exercise), and monocytes (12 h post-exercise) in the shorter inter-set rest condition. Altogether, increased inflammatory process due to increased muscle damage, increased pro-inflammatory cytokines (IL-1β, TNF-α, and GM-CSF) mainly after 6 and 12 h. Following the aforementioned increase, an increase in anti-inflammatory cytokines (IL-5, IL-6, and IL-10) was observed (6, 12, and 24 h). In accordance with these findings, the shorter rest period length triggered significantly higher RPE values compared to the longer rest condition for both volume-equated training protocols.

The level of serum CK and LDH can be elevated due to muscle tissue damage as a consequence of high-intensity training (Rodrigues et al., 2010). These increases may be a response to metabolic and mechanical stimuli (Brancaccio et al., 2007). One of the mechanisms may be related to local tissue damage with degeneration and sarcomeric fragmentation between the *Z* discs (Brancaccio et al., 2007). Another mechanism that seems to be associated with this damage is metabolic exhaustion of muscle fibers, which in turn presents a decrease in membrane resistance after an elevation in free internal calcium ions, as well as the activation of the potassium channels, occurred by the reductions in cellular energy reserves (Fink and Luttgau, 1976). Therefore, the response pattern of these enzymes (CK and LDH) suggests an appropriate indicator of the intensity of a previously performed exercise (Bessa et al., 2016). Therefore, it is plausible to interpret that different manipulations in RE variables can trigger distinct biological responses (Helms et al., 2020). Nevertheless, other studies have indeed focused on the impact of rest interval manipulation on muscle tissue damage and immune responses (Rodrigues et al., 2010; Evangelista et al., 2011; Machado et al., 2012; Gerosa-Neto et al., 2016; Rossi et al., 2016). However, these present great variations in blood time-point choices, populations, non-equalized vs. equalized volume and exercise schemes, relative loads, and nutritional controls. All this variability, unfortunately, prevents a consensus regarding post-exercise muscle tissue damage and immune responses patterns in acute response to distinct inter-set lengths.

Rodrigues et al. (2010) compared CK and LDH concentrations at multiple post-exercise time-points (>24 h) after RE sessions with different inter-set intervals. The two experimental sessions consisted of three sets with loads of 80% of 1-RM until concentric failure, which caused a 24% larger volume for the longer interval (non-equalized method). There were no significant differences in CK and LDH concentrations at any post-exercise assessment between the different intervals. Other authors observed the effects of different rest intervals on CK and LDH in sessions with 10-RM loads until concentric failure (Machado et al., 2011). The intervals stipulated in this experiment were 1-, 1.5-, 2-, and 3-min between sets and exercises. The CK and LDH were significantly elevated after 24 and 72 h in all sessions and without differences between the distinct intervals. When comparing these studies (Rodrigues et al., 2010; Machado et al., 2011) with the present experiment, there is an evident methodological difference, mainly at the proposed attempt to analyze the interval time between sets without any control of other variables as volume or intensity.

It is important to underline, that in practical terms different rest periods are chosen to allow full (longer interest periods) or incomplete recovery (shorter interest periods) between sets. In the case of longer rest periods, this avoids muscle impairments, with the consequence to counteract load decreases caused by fatigue (Schoenfeld et al., 2016), which may occur if using interest length shorter than 2 min (Matos et al., 2021). Despite perceived exertion may result increased with lower-rest intervals (Scudese et al., 2015), a progressive load decrease along with subsequent sets determines a decrease in both total volume and intensity (Matos et al., 2021), which could result in lower gains in terms of hypertrophy and strength (Schoenfeld et al., 2015, 2019).

In another investigation (Evangelista et al., 2011) which compared the differences between two distinct rest interval lengths (1- vs. 3-min) on volume, muscle damage, and muscle soreness, the subjects performed an experimental protocol consisting of three sets of biceps exercise with 40% of maximal voluntary contraction. The results showed that individuals who performed the exercises with the longer rest intervals performed more volume (as expected). Still, there were no differences for CK (24 and 48 h after exercise) and muscle soreness between groups, probably due to the low intensity performed. Similarly, Machado et al. (2012) examined the values of CK activity after RE sessions in subjects that the authors classified as possessing high, medium, or low responsiveness to the exercises. Individuals classified with high and medium responsiveness demonstrated an elevated CK activity after the 1-min interval session compared to the 3-min interval session. However, it is important to note that the blood examinations were restricted to 24 and 48 h post-exercise, and the training routine consisted of only one single-joint exercise (bicep curl).

By analyzing the previous studies’ methodology, it is safe to state that our experiment is original regarding the training volume equalization and the meticulously tailored timing of CK and LDH blood examinations. More specifically, our results allowed to observe that the shorter inter-set period potentiated the muscle damage markers responses. It should also be noted that the main focus of the present study was the initial and rapidly (up to 24 h) curve identification of CK and LDH responses, demonstrating an early post-exercise sensibility of those biomarkers. Moreover, the CK increase had its onset as early as 6 h post-exercise for the 1-min interval, and the LDH showed its peak around the 12 h post-exercise assessment. These data bring to light new evidence that the majority of the previous investigations might have missed the sensitivity window for those responses.

Regarding the pro- and anti-inflammatory process resulting from the RE responses, shortly after the muscle damage occurrence, there was leukocyte mobilization followed by its migration to the injured muscle tissue (Peake et al., 2006). The neutrophils and monocytes, the cellular types mobilized in greater magnitude at different moments in the process of muscle tissue remodeling, are responsible for the reduction of the damaged muscular tissue and the release of pro- and anti-inflammatory cytokines.

Several studies on the inflammatory responses to RE (Peake et al., 2006; Phillips et al., 2010) showed that the total training volume (rather than intensity) directly influences pro-inflammatory interleukins up to 12 h after training. Smith et al. (2000) and Hirose et al. (2004) observed important modifications for pro-inflammatory interleukins in assessments performed up to 72 h after exercise even with low training volumes. Despite evidence exists regarding manipulation of RE volume, little is known regarding inter-set differences and inflammatory responses. An investigation by Kraemer et al. (1996) in 1996 examined the impact of exercise-induced circulating plasma cortisol elevations and leukocyte counts with different inter-set lengths. Venous blood samples were obtained pre, during exercise, and 5-min after exercise. There were no significant variations in leukocyte differential counts. Probably, due to the very close assessment point, 5 min post-exercise. Though, seeking to determine the effect of distinct rest intervals on leukocyte levels during moderate-intensity RE, Mayhew et al. (2005) performed a study where nine men completed exercise sessions with 1 vs. 3 min inter-set intervals. Blood was collected at rest, immediately after, 60-, and 90-min post-exercise, and the leukocyte concentration was analyzed. Increased lymphocytosis and monocytosis were observed after the 1-min inter-set length protocol but not after the 3-min inter-set length protocol. Although the studies by Kraemer et al. (1996) and the more recent of Mayhew et al. (2005) have verified different inter-set length and their responses to leukocyte count, important limitations, such as the timing of the blood sample collection may have compromised the outcomes retrieved. In the present study, the leukocyte count was aligned with the CK and LDH assessments during the process of tissue damage (up to 24 h after exercise). In such consideration, possibly after muscle damage in response to high-intensity RE, occurred an important leukocyte mobilization and invasion (mainly neutrophils) to the injured tissue alongside with a significant monocyte elevation.

More recently, a two-part experiment (Gerosa-Neto et al., 2016; Rossi et al., 2016) aimed to verify the influence of a very short (30-s) and moderate (90-s) inter-set length on performance, inflammatory, and metabolic responses in healthy adults and recreational weightlifters. In part 1, eight healthy subjects performed two exercises at 70% of 1-RM; and in part 2, the procedures were repeated; however, with 90% of 1-RM. Both conditions each with the different inter-set lengths. The TNF-α, IL-6, and IL-10 were assessed at baseline, immediately after exercise, 15 and 30 min post-exercise. The authors concluded that exhaustive and heavy strength exercises conducted with different inter-set length decreased performance; however, an augmented inflammatory and metabolic response were found only in the longer 90-s interval. Despite similarities in inter-set length are observed when comparing these studies to our experiment, differences occur in the timing of the post-exercise blood assessments and the volume equalization.

Other finding of this investigation, is that related to perceived exertion of the participants. We demonstrated significant increases in RPE with the progression of the sets for both exercises, with the highest values found for the shorter 1-min inter-set length. To date, only two investigations compared different rest conditions on the RPE responses in submaximal exercises, both observing that the shorter rests, increased RPE responses (Scudese et al., 2015, 2016). Other experiments focused on the RPE influences of different rest intervals between sets in distinct types of exercise (Senna et al., 2011) and near-maximum loads zones (Scudese et al., 2015; Senna et al., 2016).

Limitations to our study are the relatively small sample size, since not many participants who met all the required inclusion criteria were willing to participate due to the repeated blood sample collection. Further, our evaluation up to 24 h post-exercise only is a further limitation as muscle damage and inflammation typically occur up until 48 h post-exercise. However, it was aim of this study to evaluate the curve response within the first 24 h. The inclusion of a third 5-min inter-rest condition could have provided further confirmation regarding the data included for the 3-min inter-rest length. Further consideration could be that the intensity used across the two protocols was 85% of 10RM, which may not be of sufficient intensity to produced muscle damage with long rest periods and may have produced different responses compared to repetition maximum training.

## Practical Applications

This study brings a new approach to the way muscle damage and inflammatory response are investigated after a resistance training routine. Our results suggest that when equalized for training volume, a shorter inter-set length promotes a considerably greater damage to muscle tissues, as well as a longer duration of the inflammatory process of this tissue. Based on these findings, specifically on the durable inflammatory augmentation observed with the shorter inter-set length, practitioners with the intent to promote hypertrophy or local muscle endurance development should consider implementing shorter inter-set lengths when using a similar type of training method. Other important aspect to consider is that shorter inter-set periods allow shorter workouts, which in turn improve training efficiency.

This data might contribute to future recommendations focused on different goals requiring considerable muscle tissue damage and inflammatory response for its optimization. These results are applicable and limited to the specific exercises, inter-set length, and load examined. However, we strongly recommend that future studies should evaluate distinct exercise schemes, other load ranges, types of rest period (active vs. passive), and whole-body training sessions.

## Data Availability Statement

The raw data supporting the conclusions of this article will be made available by the authors, after reasonable request.

## Ethics Statement

The studies involving human participants were reviewed and approved by the Federal University of State of Rio de Janeiro ethics committee (CAAE 63803717.2.0000.5285). The patients/participants provided their written informed consent to participate in this study.

## Author Contributions

All authors listed have made a substantial, direct, and intellectual contribution to the work and approved it for publication.

## Funding

This study was financed in part by the Coordenação de Aperfeiçoamento de Pessoal de Nível Superior – Brasil (CAPES)–Finance Code 001.

## Conflict of Interest

The authors declare that the research was conducted in the absence of any commercial or financial relationships that could be construed as a potential conflict of interest.

## Publisher’s Note

All claims expressed in this article are solely those of the authors and do not necessarily represent those of their affiliated organizations, or those of the publisher, the editors and the reviewers. Any product that may be evaluated in this article, or claim that may be made by its manufacturer, is not guaranteed or endorsed by the publisher.
